# Speckle Filtering of GF-3 Polarimetric SAR Data with Joint Restriction Principle

**DOI:** 10.3390/s18051533

**Published:** 2018-05-12

**Authors:** Jinwei Xie, Zhenfang Li, Chaowei Zhou, Yuyuan Fang, Qingjun Zhang

**Affiliations:** 1 National Laboratory of Radar Signal Processing, Xidian University, Xi’an 710071, China; jerryxie9@gmail.com (J.X.); zcwlzyz@sohu.com (C.Z.); fangyuyuan94@163.com (Y.F.); 2 Beijing Institute of Spacecraft System Engineering, China Academy of Space Technology, Beijing 100094, China; zhangqj@cast.cn

**Keywords:** GF-3, speckle filtering, polarimetric synthetic aperture radar (PolSAR), restriction principle

## Abstract

Polarimetric SAR (PolSAR) scattering characteristics of imagery are always obtained from the second order moments estimation of multi-polarization data, that is, the estimation of covariance or coherency matrices. Due to the extra-paths that signal reflected from separate scatterers within the resolution cell has to travel, speckle noise always exists in SAR images and has a severe impact on the scattering performance, especially on single look complex images. In order to achieve high accuracy in estimating covariance or coherency matrices, three aspects are taken into consideration: (1) the edges and texture of the scene are distinct after speckle filtering; (2) the statistical characteristic should be similar to the object pixel; and (3) the polarimetric scattering signature should be preserved, in addition to speckle reduction. In this paper, a joint restriction principle is proposed to meet the requirement. Three different restriction principles are introduced to the processing of speckle filtering. First, a new template, which is more suitable for the point or line targets, is designed to ensure the morphological consistency. Then, the extent sigma filter is used to restrict the pixels in the template aforementioned to have an identical statistic characteristic. At last, a polarimetric similarity factor is applied to the same pixels above, to guarantee the similar polarimetric features amongst the optional pixels. This processing procedure is named as speckle filtering with joint restriction principle and the approach is applied to GF-3 polarimetric SAR data acquired in San Francisco, CA, USA. Its effectiveness of keeping the image sharpness and preserving the scattering mechanism as well as speckle reduction is validated by the comparison with boxcar filters and refined Lee filter.

## 1. Introduction

GF-3, the first C-band multi-polarization synthetic aperture radar (SAR) in China, was launched in August 2016. It has twelve different imaging modes, including spotlight mode, ultra-fine strip mode, scanSAR mode, full polarized strip mode, etc. The resolution of the spotlight mode can reach as high as 1 m, which can rank first in the C-band multi-polarized SAR systems all over the world [1]. For the satellite’s advantage of working all-weather and all-day, these imaging modes can provide an abundant supply of data for various applications of the global observation, such as mapping the terrain on a large scale, monitoring the forestry resource for the management of exploiting the timber, as well as conservation of the wild life, monitoring the marine environment for meteorological research or oceanographical research, or other techniques [2]. According to the open studies, the exploration about GF-3 polarimetric SAR data has aroused widespread interests. Yin et al. [3] provided an analysis on the physical scattering mechanism of GF-3 polarimetric SAR data and showed that the data has great potential for target characterization. Shao et al. [4] developed the method of strong wind retrieval from the cross-polarization GF-3 SAR data. Chang et al. [5] had an experiment on the calibration of residual polarimetric distortion for channel imbalance and crosstalk of GF-3 polarimetric SAR images and improved the quality of image greatly. Dong et al. [6] investigated the quality of GF-3 polarimetric SAR for classification purpose with the incorporation of XGBoost and polarimetric spatial information. Liu et al. [7] developed an unsupervised change detection method with time-series of GF-3 polarimetric SAR images and showed the method not only can suppress the speckle noise greatly but also have good performance in change detection. It is obvious that the usage of polarimetric SAR data is of great importance in the ground surface observation or ocean surveillance, especially for the ground parameters inversion and ground classification. While an inevitable processing for the majority of applications of polarimetric SAR data is to acquire the covariance or coherency matrices with high accuracy, which requires speckle noise reduction. Thus, it is of great significance to study the speckle filtering of GF-3 polarimetric SAR data.

Speckle is an inherent phenomenon in SAR image because of the coherent superposition of waves reflected from a number of elementary scatterers in a single pixel [8]. Speckle filtering of polarimetric SAR data is always an essential step for the SAR image interpretation. The improper filtering of speckle will lead to an inappropriate illustration of media’s scattering mechanisms, especially for the entropy from polarimetric decomposition and the degree of polarization. The entropy is zero in a single look complex plarimetric SAR data, while it increases with number of pixels because of the inclusive of different scattering mechanisms. For example, with the increase of pixels, that is, the increase of filter window size, more heterogeneous pixels which may indicate different scattering mechanisms are included in the window of the boxcar filter, thus leading to high entropy [9]. Nowadays, with the development of the polarimeric SAR platform, a variety of speckle filtering approaches are proposed for the particular data. 

Novak and Burl first explored the speckle suppression of polarimetric SAR data. A polarimetic whitening filter (PWF) [10] was derived by combining all elements of the covariance matrix optimally and a single, speckle-reduced image is produced on the EMISAR’s single look polarimetric SAR data. Afterward, Liu et al. [11] extended the PWF for multi-look in the form of covariance or coherency matrix and obtained a filtered image, while the deficiency of this method is that the polarimetry information is lost because it comes out only a combined intensity image. To reduce the speckle of the three polarimetric channels of HH, VV, and HV images, Lee et al. [12] proposed a linear speckle filter on the hypothesis that the speckle noise model adapts to the multiplicative noise model. Based on this hypothesis, Lin et al. [13] developed a vector filter by using the minimization of the mean square error. Nevertheless, it cannot filter all the elements of the covariance or coherency matrix. Goze and Lopes [14] extended the approach for exclusive single look imagery to cover all elements of the covariance. For the reason that the depiction of multiplicative noise model for the off-diagonal terms of the covariance is controversial, Lopes and Sery [15] developed several filtering approaches to account for the texture variation. All the filters aforementioned exploited the degree of statistical independence within the linear polarization channels. The crosstalk between channels cannot be addressed properly and the polarimetric information is not preserved well [9]. As for the simplicity and computational efficiency, we find that the boxcar filter is still the most commonly used approach to handle with the speckle reduction of poalrimetric SAR imagery, especially for the application of parameter inversion from homogeneous media. While it is at the cost of degrading the resolution and spatial details, and considerable square imprints emerge when both the targets show point scattering and the window size is large. 

In order to cope with the issues above, Lee et al. [16] proposed a new approach in polarimetric SAR filtering. The minimum mean square error (MMSE) was used to both the diagonal and the off-diagonal elements of the covariance or coherency matrix, which was named as Lee Filter. Subsequently, the edge-aligned windows were first employed to preserve the image details, and the filter was defined as Refined Lee filter [17]. To come up with this method, three basic principles were taken into account as follows: (1) all the elements in the covariance or coherency matrix have to be filtered independently in the spatial domain to avoid cross-talk; (2) each term of the covariance or coherency matrix should be filtered in the same edge-aligned window, which is similar to multi-look processing, to preserve polarimetric properties; and (3) the filter is self-adaptive to weight the covariance or coherency matrix of the center pixel with the mean value from selected neighboring pixels. This principle is to preserve point targets, as well as speckle elimination. Though the three principles are considered, the capability of keeping the edge sharpness is very limited in the heterogeneous regions, such as the streets, the city blocks, the bright point scatterers, etc. The streets always show linear features and the single point scatterers cambered features. While all the windows in Refined Lee filter still have the square brims. That is to say the edge-aligned windows of the Refined Lee filter do not match very well to the edge signature when the scene becomes complicated.

In [18], an improved sigma filter was proposed for speckle filtering of SAR imagery. The statistic characteristics, which were first taken into consideration by sigma filter [19,20] based on the simple concept of two-sigma probability, were fully utilized. The filter is very effective in eliminating the deficiencies of sigma filter, such as bias in the estimates, unfiltered black pixels, and smearing of the strong targets. In this processing, the sigma range was redefined based on the theoretical speckle distribution to remove the bias of mean values. The MMSE estimator was incorporated to address the problem of leaving out black pixels and depressing strong reflective scatterers. In [21], the improved sigma filter was extended to polarimetric SAR imagery. In the procedure, the speckle filtering was applied only to distribution media, and the strong targets were leaved unfiltered. The estimation of priori mean and sigma range were using the pixels selected by all the three polarizations corresponding to the specular, double bounce and volume scattering mechanisms. This implementation was addressed to account for the preservation of scattering mechanisms. Whatever estimations by the coherency or covariance matrix are carried out, the polarimetric properties are only from the diagonal elements of the matrix. The off-diagonal elements from the matrix do not contribute so much to the polarimetric information preservation.

In recent years, a novel idea of despeckling algorithm for polarimetric SAR data has been proposed [22,23,24,25]. The basic idea is to take advantage of image self-similarity. The main principle is to select homogeneous pixels in a large-scale area (always 15 × 15 pixels) in the filtering process. In [22], the homogeneous pixels are selected by comparing a 3 × 3 patch in a 15 × 15 searching window based on the complex Wishart distribution, and then the filtering process is performed by summing up the homogeneous pixels with weights according the test statistics values. The algorithm can preserve strong point targets, edges and texture feature while suppressing speckle. Nevertheless, the polarimetric scattering mechanisms are not analyzed so much. In [23], the non-local filter is extended for denoising polarimetric or interferometric SAR images. The algorithm is fully automatic and handles single and multi-look images and offers a flexible frame work for resolution-preserving speckle reduction. In [24,25], both the speckle filters are scattering-based nonlocal means. With the performance of speckle reduction and detail preservation they are even able to preserve scattering mechanisms of the illuminated surface. One dominant deficiency of all these filters is that they are very time-consuming because of the complex process procedures.

Besides the filters mentioned above, a set of polarimatric SAR speckle filters are presented according the open literatures, such as the scattering model-based filter (SMB) [9], the trace-based filter (TBF) [26], the subspace filtering of Mueller matrix (SFMM) [27], etc. For the SMB, only those pixels in a square window corresponding to the same scattering mechanism, which are obtained from the Freeman-Durden decomposition, as the central pixel are considered. Pixels with similar dominant scattering mechanism are grouped into clusters and then, a Local Linear Minimum Mean Square Error (LLMMSE) filtering is applied using only pixels in the same cluster. This filter is effective in speckle reduction as well as preserving strong point target signatures perfectly. While the algorithm needs to classify the PolSAR image first, about which the accuracy of classification is still a question under some circumstances. For the TBF, it is an iterative partial differential equations (PDE) filter that is equivalent to a local convolution by oriented Gaussian filters. The shapes of the local Gaussian filters are derived from the local image structure, through the structure tensor. The amplitudes of all the covariance matrix terms are considered to construct the structure tensor. The filter can retain a good performance between a high degree of smoothing and detail preservation. While the deficiency is that it needs some efficient controls of the filtering process and the number of iteration is difficult to decide. As for the SFMM, the first step of process procedure is to establish a local polarimetric feature matrix composed of the normalized Mueller coefficients. The matrix is named the parameter space and consists of two orthogonal subspaces which are the signal subspace and the noise subspace, respectively. A subspace decomposition (PCA) is performed and the local polarimetric information is then reconstructed from only the significant PCA coefficients. In this way, edges of different kinds of targets are preserved for a good performance of noise reduction. While deficiency for this method is that the signal subspace and the noise subspace we choose from the decomposition result cannot keep completely orthogonal, thus leading to a leak of noise into the signal subspace, i.e., the despeckling does not perform very well. In this paper, we first explore the speckle filtering performance of GF-3 polarimetric SAR imagery in strip imaging mode. In order to mitigate the degrading of the spatial resolution, and to preserve the polarimetric properties on a large extent, in addition to the speckle reduction, three principles are introduced to meet with the requirement above. Then a joint restriction principle is proposed for the polarimetric SAR filtering processing. The remainder of this paper is organized as follows: Section 2 describes the three restriction principles. Section 3 gives the details processing procedure of joint restriction Principle. In Section 4, the experiments on GF-3 polarimetric SAR data are conducted to validate the effectiveness of the proposed method. The comparisons with the common and typical filtering algorithms as well as the discussion are carried out in this section. Conclusions are drawn in Section 5. 

## 2. Method

### 2.1. Polarimetric Covariance and Coherency Matrices

Polarimetric radar obtains the complex scattering matrix S of a medium with the combination of horizontal or vertical polarization between the transmitting and the receiving channels [28]. Based on the hypothesis of reciprocal backscattering case, $S_{hv}=S_{vh}$, the scattering matrix can be expressed vectorially by the Lexicographic basis or by the Pauli basis [29] as:

(1) $$k_{l}={\left[S_{hh},\;\sqrt{2}S_{hv},\;S_{vv}\right]}^{T}$$

(2) $$k_{p}=\frac{1}{\sqrt{2}}{\left[S_{hh}+S_{vv},\;S_{hh}-S_{vv},\;2S_{hv}\right]}^{T}$$

where $S_{xy}$ is the element of S, the subscript h and v denote the horizontal and vertical wave polarization states, respectively. The coefficient $\sqrt{2}$ or 2 is to ensure the consistency in the span (total power) computation. The superscript T denotes the matrix transpose.

Polarimetric SAR scattering characteristics of imagery are always obtained from estimation of the second order moments for multi-polarization data, that is, the estimation of covariance or coherency matrix [30]. Based on the scattering vector, the matrices can be expressed in following form:

(3) $$C=k_{l}k_{l}^{H}=\left[\begin{array}{ccc} |S_{hh}{|}^{2} & \sqrt{2}S_{hh}S_{hv}^{*} & S_{hh}S_{vv}^{*}\\ \sqrt{2}S_{hv}S_{hh}^{*} & 2|S_{hv}{|}^{2} & \sqrt{2}S_{hv}S_{vv}^{*}\\ S_{vv}S_{hh}^{*} & \sqrt{2}S_{vv}S_{hv}^{*} & |S_{vv}{|}^{2} \end{array}\right]$$

(4) $$T=k_{p}k_{p}^{H}=\frac{1}{2}\left[\begin{array}{ccc} |S_{hh}+S_{vv}{|}^{2} & (S_{hh}+S_{vv}){\left(S_{hh}-S_{vv}\right)}^{*} & 2(S_{hh}+S_{vv})S_{hv}{}^{*}\\ (S_{hh}-S_{vv}){\left(S_{hh}+S_{vv}\right)}^{*} & |S_{hh}-S_{vv}{|}^{2} & 2(S_{hh}-S_{vv})S_{hv}{}^{*}\\ 2S_{hv}{\left(S_{hh}+S_{vv}\right)}^{*} & 2S_{hv}{\left(S_{hh}-S_{vv}\right)}^{*} & 4|S_{hv}{|}^{2} \end{array}\right]$$

where the superscript H denotes the complex conjugate transpose of a vector and ∗ the complex conjugate. The span (or total power) can be obtained through the addition of diagonal elements from covariance or coherency matrix:

(5) $$\mathrm{span}=|k_{l}{|}^{2}=|k_{p}{|}^{2}=|S_{hh}\;{|}^{2}+2|S_{hv}{|}^{2}+|S_{vv}{|}^{2}$$

Due to the extra-paths that signal reflected from separate scatterers within the resolution cell has to travel, the speckle noise always exists in SAR image and has a severe impact on the scattering performance, especially on single look complex images. The speckle filtering of polarimetric SAR data is to depress the impact of speckle by averaging several neighboring one-look pixels with the special restriction principles. Thus, we form filtered covariance matrix and coherency matrix shown as:

(6) $$C=\frac{1}{N}\sum_{i=1}^{N}k_{l}^{i}k_{l}^{i}{}^{H},\;T=\frac{1}{N}\sum_{i=1}^{N}k_{p}^{i}k_{p}^{i}{}^{H}$$

where N is the number of the pixels chosen from the homogeneous area or the number of the nominal multilook. In fact, this form is under the assumption of statistical ergodicity and stationarity, and the multilook processing is substituted by spatial averaging of N independent samples for the maximum likelihood estimation (MLE) [31]. 

In order to achieve high accuracy of covariance or coherency matrices, three aspects are taken into consideration, the first one to be considered is that the edges and texture of the scene are distinct after speckle filtering. This principle is in agreement with maintaining of the spatial details, especially in case of point scatterers [32]. The second one is that the statistic distribution should be considered to ensure the similar radiometric amplitude [33], and the additional principle is that the polarimetric scattering properties from each elements of covariance matrix or coherency matrix should be exploited on a large extent. As for the second moments of multidimensional SAR data, the covariance and the coherency matrices are related by similarity transformations [34]. Implicitly, the filtered matrix can be acquired from one another. Because the coherency matrix is formulated closer to the physical scattering processing than the covariance matrix, we only concentrate on the estimation of coherency matrix in the following speckle filtering study. However, the algorithm can be applied directly to covariance matrix under a slight modification.

### 2.2. Morphological Consistency

As for the most commonly used speckle filter of SAR imagery, the boxcar can smear edges severely in heterogeneous regions. It may present a large quantity of square imprints surrounding the strong point scatterers, which results in inconformity with the real structure [9]. The Refined Lee filter can lower this impact effectively by using edge-aligned windows in eight patterns [35]. The edge direction is determined by a simple 3×3 edge-mask from the means of the sub-windows. Then the multiplicative model is applied and the weight is compute by the pixels from the selected edge-aligned window. Though the edge direction is considered, all the windows in Refined Lee filter still have the square brims. Pixels in the window are considered to be homogeneous. Actually, all the natural targets in SAR images are not with signature of square edges, such as the streets, the city block, the bright point scatterers, etc. The streets or blocks always show linear brims and the single point scatterers cambered brims. That is to say the edge-aligned windows of Refined Lee filter do not fit very well to the edge signature when the scene becomes complicated, thus causing a large errors.

To cope with the issue above, we come up with a group of new windows, which are very similar to the edge-aligned windows of Refined Lee filter. Likewise, to determining the direction of the new windows, the initial boxcar window is still divided into 3×3 subwindows and the four edge masks are applied to means of the subwindows [36]. Here the four edge masks are:

$$\left[\begin{array}{ccc} -1 & 0 & 1\\ -1 & 0 & 1\\ -1 & 0 & 1 \end{array}\right]\left[\begin{array}{ccc} 1 & 1 & 1\\ 0 & 0 & 0\\ -1 & -1 & -1 \end{array}\right]\left[\begin{array}{ccc} 1 & 1 & 0\\ 1 & 0 & -1\\ 0 & -1 & -1 \end{array}\right]\left[\begin{array}{ccc} 0 & 1 & 1\\ -1 & 0 & 1\\ -1 & -1 & 0 \end{array}\right]$$

**Figure 1:**
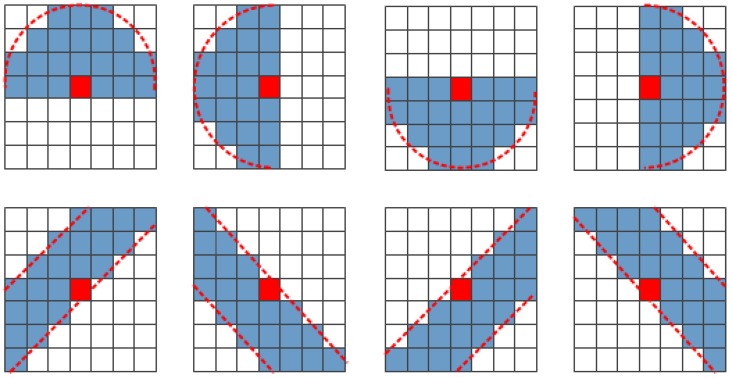
The morphological windows with cambered brims (**upper side**) and linear brims (**lower side**).

The new windows are designed on two types. One is for the single point scatterers, and the other is for streets or city blocks. The former types hold the brims close to cambered signature, while the latter close to linear signature. Figure 1 shows the features of our proposed windows. Pixels where the outline is close to a semicircle are selected for homogeneity in a boxcar window to fit with the structure of point scatter, and pixels with outline closing to line are selected to fit the structure of streets or city blocks. The size of boxcar can be 7×7, 9×9, 11×11, or larger. Here we take 7×7 window for example. The upper side of Figure 1 presents four windows with asymptotic cambered brims (red dashed curves), where the dome direction are 90°, 180°, 270° and 0°. The lower side of Figure 1 shows four windows with asymptotic linear brims (red dashed lines). Nevertheless, the line directions are mainly 45° and 135° respectively for the reason of symmetry. All the pixels selected for filtering are colored in light blue excluding the center pixel (object pixel) with red color.

As these windows can preserve the spatial details more precisely, we treat this speckle filtering restriction as a morphological consistency principle and the windows are called morphological windows. We abbreviate this algorithm as MCPF (morphological consistency principle filter), which is deemed a critical restriction in the speckle filtering of polarimetric SAR imagery. Here we describe the processing of selecting the morphological window briefly in few steps:

- The 7 × 7 boxcar window is divided into nine subwindows. The subwindows are 3 × 3 in size and have overlaps with each other.
- The means of the span images of all the subwindows are evaluated to form a 3 × 3 mean-window.
- The edge masks are applied into the mean-window. The horizontal and the vertical directions of the edge masks are to determine the direction of morphological windows with cambered brims, and the 45° and 135° directions with linear brims. For example, if the maximum absolute value of summation of all the elements which are formed by the Hadamard product of the 3 × 3 mean-window and the edge masks corresponds to the first edge mask, the windows with cambered of dome-direction 180° and 0° are selected, and if the maximum absolute value corresponds to the third edge mask, the windows with linear direction of 45° are selected. Thus, each of the edge masks can select two morphological windows in symmetry.
- One of the two morphological windows in symmetry is selected based on the closeness of the center submean to the submeans in the pattern direction.

### 2.3. Statistic Characteristics Restriction

Though the morphological consistency should be considered, the statistic characteristics of speckle are also very essential for the preservation of structure features in the speckle reduction, especially for the distributed media. It is obvious that the number of direction for morphological windows proposed in Section 2.2 is very limited. No matter the windows with cambered brims or the windows with linear brims, there are only four directions in the templates. To allow the selection of new windows to have the similar processing with Refined Lee filter, we do not consider the window with linear brims both in vertical and horizontal directions at all. In some cases, neither the windows could fit very well with the targets, nor each pixel in the morphological windows has the same statistic distribution in the reflectance. Therefore, it is necessary to take the statistic characteristics into consideration, as well as the morphological consistency. 

In [21], an effective algorithm which was an extension of the improved Lee sigma filter was proposed to investigate the speckle characteristics for speckle filtering of polarimetric SAR imagery. In this section, we have no intention for developing a new algorithm. The main objective is to describe this algorithm briefly and try to apply this restriction principle to our processing of speckle filtering.

**Figure 2:**
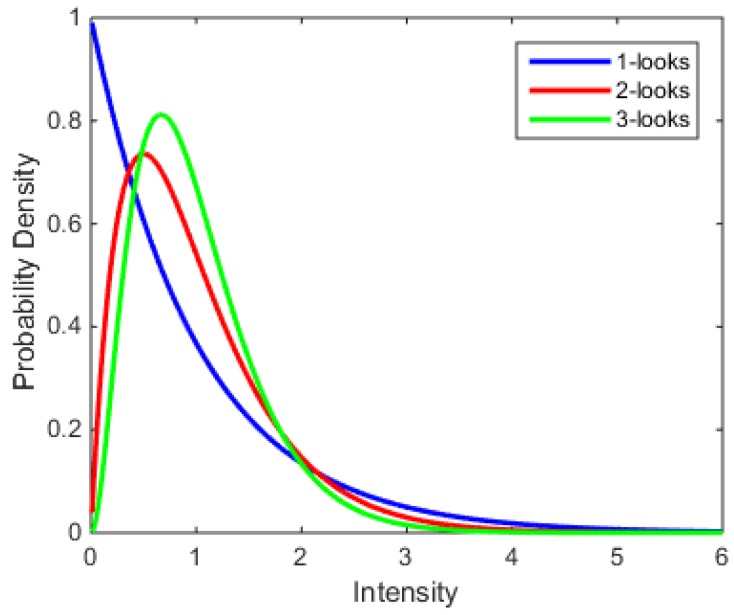
Intensity pdfs of one-, two-, and three-look data.

The improved Lee sigma filter was devised to overcome several deficiencies as follows: (1) for single look amplitude and intensity SAR data, the probability distributions follow the Rayleigh and the negative exponential distribution which are far from symmetrical. While the sigma range in Lee sigma filter follows Gaussian distribution, which fails to maintain the mean value and produces biased results; (2) most algorithms assume that there exists a large number of random scatterers within a resolution. Each pixel is averaged by neighboring pixels, thus, strong hard targets are blurred and their power is reduced; (3) pixels with low intensities have a very small sigma ranges. In an extreme case, the sigma range is near to zero. Consequently, dark spotty pixels are not filtered. To address these issues, the improved sigma Lee filter took some strategies and then was extended to polarimetric SAR data. For the new algorithm, the N-look intensity pdf is described by [18]:

(7) $$p(I)=\frac{n^{n}I^{n-1}}{(n-1)!{\bar{I}}^{n}}\exp(-nI/\bar{I}),\;I\;\geq 0$$

where n is the number of looks. The mean of intensity is $M(I)=\bar{I}$ and the variance is $Var(I)={\bar{I}}^{2}/n$. The one-, two-, and three-look pdfs with M(I)=1 are given in Figure 2 [18]. Notably, from Figure 2 we can see that all these curves are far from symmetric.

In the process of selecting pixels included into the speckle filtering, the intensity of pixels is restricted into a sigma range of $\left(I_{1},I_{2}\right)$, where the boundary ensures that the probability is equal to a given sigma value, and the mean of intensity is still $\bar{I}$. The conditions show as follows:

(8) $$\xi =\int_{I_{1}}^{I_{2}}p(I)dI$$

(9) $$\bar{I}=\frac{1}{\xi }\int_{I_{1}}^{I_{2}}I\cdot p(I)dI$$

To ensure a unique solution for $I_{1}$ and $I_{2}$, a numerical search technique is applied. We use the intensity case with $\bar{I}=1$ as an example. In the first iteration, we set $I_{1}^{\left(1\right)}=0.5$, and $I_{2}^{\left(1\right)}$ is computed from (8). The mean value ${\bar{I}}^{\left(1\right)}$ is computed from (9). If ${\bar{I}}^{\left(1\right)}<1$, $I_{1}$ is subtracted by a small increment (for example, 0.001) or else added by a small increment. The iteration continues until convergence with ${\bar{I}}^{\left(J\right)}=1$. Because of the multiplicative nature of the speckle, it is not necessary to compute sigma range for all mean values. If we set the mean $\bar{I}=1$, and compute $\left(I_{1},I_{2}\right)$, the sigma range for all mean values can be obtained by simply multiplying $\left(I_{1},I_{2}\right)$ by the mean value under a given sigma value. For the case of the mean value of 1, the sigma ranges $\left(I_{1},I_{2}\right)$ of the intensity SAR data computed for sigma values between 0.5 and 0.95 for one-look, are shown in Table 1. For any mean value $\tilde{x}$, the new sigma range can be easily computed from the table as $\left(I_{1}\tilde{x},I_{2}\tilde{x}\right)$.

An important step in this processing is estimation of the mean value of intensity $\tilde{x}$. The Lee sigma filter assumed the center pixel of the filtering window as the priori mean $\tilde{x}$. This produced the isolated dark pixel when the intensity is low enough and the sigma range is close to zero. To cope with this issue, a 3 × 3 window can be used to calculate an average as $\tilde{x}$. However, it may cause a slight edge blurring problem. While the improved sigma filter applied the MMSE [16,35] in the 3 × 3 window to estimate the priori mean $\tilde{x}$, which effectively retain fine details as well as reducing the isolated dark pixels. The MMSE filter based on multiplicative noise model has the form

(10) $$\hat{x}=(1-b)\tilde{z}+bz$$

with:

(11) $$b=\frac{Var(x)}{Var(z)}$$

(12) $$Var(x)=\frac{Var(z)\;-{\tilde{z}}^{2}\eta_{v}^{2}}{1+\eta_{v}^{2}}$$

where the $\tilde{z}$ is local mean and Var(z) is local variance, and both of them are computed using the 3 × 3 window. Var(x) is the variance of x computed by (12). The parameter $\eta_{v}$ is the standard deviation of multiplicative noise and is the function of number of looks. For one-look intensity data $\eta_{v}=1$, and for N-look SAR data, $\eta_{v}=\sqrt{1/n}$. It is worth mentioning, the standard deviation value needs to be adjusted when using it to filter the pixels in a sigma ranges. Then the prior mean $\tilde{x}$ is calculated by (10), which is prepared for a second utilization of MMSE.

This algorithm is mainly considered an approach to retain the statistic characteristic of polarimetric SAR data, which is another restriction principle of the speckle filtering. Our purpose is to take a combination of the morphological consistency principle and the statistic characteristic principle in the processing to get a more precise preservation of the homogeneous pixels. Here we give the details about the combination. After the implantation of the MCPF algorithm, in the moving morphological windows, the strong targets pixels are detected and kept unfiltered (the details about how to detect the strong targets are in [21]). Then pixels selected by the new sigma range $\left(I_{1}\tilde{x},I_{2}\tilde{x}\right)$ are included in the filtering, where the sigma boundaries *I*_1_ and *I*_2_ are mentioned in Table 1. The priori means to establish the sigma range are obtained based on the diagonal elements of the coherency matrix, i.e., *T*_11_ and *T*_22_ corresponding the surface and double-bounce scattering mechanism, respectively. Then the pixels are selected in the morphological window within the sigma range. After pixels are selected, the span image (*span = T*_11_ *+ T*_22_ *+ T*_33_) is applied to calculate the weight for the final filtering of coherency matrix, where the MMSE is used once again. In addition, the speckle noise standard deviation $\eta_{v}$ has to be revised, because the valid range of the pdf is limited by the sigma range. The adjusted ${\tilde{\eta }}_{v}$ applied here are listed in Table 1 [21]. For simplicity, we abbreviate this algorithm as SCPF (statistic characteristics principle filter).

### 2.4. Scattering Mechanism Preservation

Another critical restriction principle is scattering mechanism preservation. When estimating the coherency matrix of each pixel in the full-polarization SAR data, it is necessary to consider the preservation of polarimetric scattering mechanism in the coherency matrix as one of the restriction principle in the speckle filtering. To maintain the polarimetric scattering information, most filters [8,9,10,37,38,39,40] mainly concentrate on the diagonal elements of the coherency matrix. Though the general scattering mechanisms, e.g., the surface, the double bounce and the volume scattering, are fully utilized, the information in the other elements of the coherency matrix are not exploited sufficiently. It is well known that, under the hypothesis of zero-mean multivariable complex Gaussian, the polarization scattering process can be fully described by using diagonal elements and non-diagonal elements of the coherency matrix [8]. To guarantee the accuracy of the consistency of scattering mechanism in the selected pixels with the true scattering characteristics, in this section, we come up with a scattering similarity factor (SSF). The index of the SSF can be treated as the level of preservation about polarimetric scattering mechanism. All the elements in the coherency matrix are taken full advantage of its polarimetric information. Assuming that the pixel to be filtered is u, and pixel to be selected into the filtering processing, excluding *u*, is *t.* The SSF is proposed depending on the definition of coherence coefficient in the interferometry as follows:

(13) $$\mathrm{SSF}=\frac{\left|p_{u}*conj(p_{t}^{T})\right|}{norm(p_{u})\times norm(p_{t})}$$

where **p** is a vector which includes all the elements of the coherency and contains pixel-dependent polarization scattering properties. The superscript *T* is the matrix transpose. The *norm* is the operation of 2-norm of a vector, and * is the inner product of two vectors. Obviously, the range of SSF is (0, 1). The larger the value of SSF the more similar the pixel *t* is to pixel *u* in the scattering mechanisms. When SSF=1, the polarization scattering properties of the two are exactly the same, and when SSF=0, the two are completely different. The vector **p** can be expressed as:

(14) $$\begin{array}{ll} p= & [|s_{hh}+s_{vv}{|}^{2},|s_{hh}-s_{vv}{|}^{2},4|s_{hv}{|}^{2},(s_{hh}+s_{vv})*conj(s_{hh}-s_{vv}),\\ & 2(s_{hh}+s_{vv})*conj(s_{hv}),2(s_{hh}-s_{vv})*conj(s_{hv})]/2 \end{array}$$

Among the elements of p, $s_{xy}$ is an element of complex scattering matrix S, which we present in the first section. For the polarization similarity selection using the above formula, since the vector p contains information of different polarization channels, it has the function of preserving polarization scattering mechanisms. We treat this restriction principle as scattering mechanism principle and abbreviate this algorithm as SMPF (scattering mechanism principle filter).

## 3. Joint Restriction Principle

Based on the previous analysis, all the three principles aforementioned (the MCPF, the SCPF and the SMPF) should be taken into consideration for a high accuracy of choosing the pixels in homogeneous areas, especially for sophisticated circumstance of polarimetric SAR data. In this section, we propose a joint restriction principle for polarimetric SAR speckle filtering. Pixels included into the speckle filtering are selected under the restriction of the three principles. The MCPF is first take into account to maintain the edge or texture of the scene, then the SCPF is applied to keep the consistency of statistic characteristics, and the SMPF is added to preserve the scattering mechanisms of the objective pixels at last. Before applying these principles, the initial moving window could be 7 × 7, 9 × 9, 11 × 11, or larger. Although the processing procedure has no difference, the larger the window size is, the more heterogeneous pixels are included into the filtering process. Also, the time expense should not be ignored during the computation process. Here we take 7 × 7 as the example, and we name the algorithm as JRPF (joint restriction principle filter) for simplicity. The procedure is listed as follows:

(1) Select morphological windows by MCPF: In order to get a similar processing procedure, a 7 × 7 window in the span image is divided into nine 3 × 3 subwindows. The means of the subwindows are computed to form a 3 × 3 mean-window. Two of the eight morphological windows are selected based on the given edge masks and the mean-window. The final direction of the morphological window is based on the closeness of the center submean with the two submeans in the edge-direction.
(2) Select pixels by SCPF in the morphological pixels chosen above: After choosing the morphological window, SCPF was operated to selected pixels to keep an identical scattering distribution in this window. A preprocessing is to detect the strong targets in the polarimetric SAR image to avoiding the blurring due to averaging of neighboring pixels. For polarimetric SAR data, point targets are always show strong reflectance in surface or double-bounce scattering corresponding to *T*_11_ and *T*_22_ in the coherency matrix. Instead of leaving the pixels of strong targets unfiltered, we filter this pixel in a 3 × 3 boxcar window. Because the rank of the coherency matrix in some applications of polarization cannot be less than 3 [41]. The other pixels to be filtered are used to estimate the sigma range. The priori means of *T*_11_, *T*_22_ and *T*_33_ are estimated by MMSE in 3×3 boxcar window with the regular standard deviation to mean ratio, respectively. Then the sigma range is determined by all the three polarizations through $I({\tilde{T}}_{ii}I_{1},{\tilde{T}}_{ii}I_{2})$ where (*I*_1_, *I*_2_) are shown in Table 1. At last, pixels within the sigma range are in the morphological window. Notably, the quantity of pixels selected out should not be less than a given number $K_{1}$ to avoid the low-rank phenomenon. Here we assume that $K_{1}=9$, which is similar to 3×3 boxcar window. The sigma value should be update if $K_{1}\leq 9$.
(3) Select pixels by SMPF in the morphological: The SSF is applied to the pixels between the object pixel and other pixels within the morphological window. Pixels are ignored when the SSF is under the threshold. For the same reason, the quantity of pixels selected out under this principle should not be less than $K_{2}$. Here we still set $K_{2}=9$, and update the threshold when $K_{2}\leq 9$.
(4) Filter the selected pixels with MMSE again: Pixels satisfying both the condition of SCPF and the condition of SMPF, that is, $K_{1}>9$ and $K_{2}>9$, are selected as the final set of pixels for filtering. The spans of these pixels are used to compute their mean, variance and the filtering weight, respectively. Finally, all the elements of the coherency matrix are filtered by MMSE.

**Figure 3:**
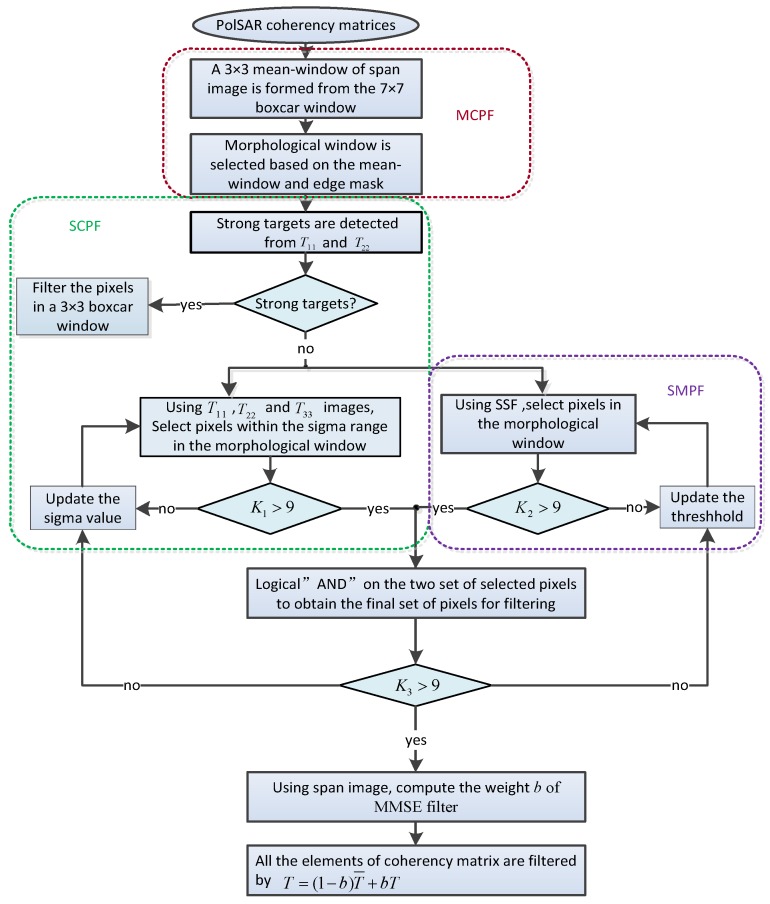
Flowchart of the proposed speckle filtering with joint restriction principle.

For clarity, the whole procedure of joint restriction principle filtering is schematically described as the flowchart in Figure 3.

## 4. Experimental Results and Discussions

**Figure 4:**
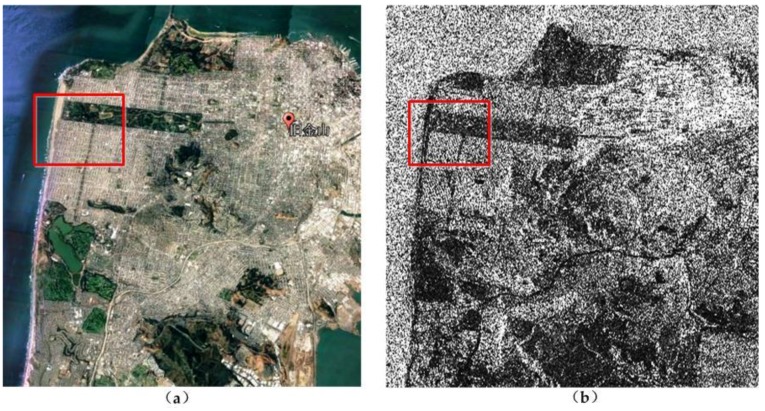
Test site selected in San Francisco, CA, USA, (**a**) Google Earth map obtained on 2 September 2017; (**b**) span image of GF-3 polarimetric SAR data collected on 15 September 2017.

To illustrate the performance of JRPF, the GF-3 polarimetric SAR data for San Francisco (CA, USA) are used as the experimental data. The acquisition mode is C-band full-polarimetric Strip I (QPSI) mode. The images are single look complex (SLC) with the resolution of 8 m. The experimental site has the main land cover of ocean area, vegetation, city blocks, street, etc. The data was acquired on 15 September 2017 on ascending passes with right looking. Figure 4 shows a Google Earth map obtained on 2 September 2017 and the span image of GF-3 polarimetric SAR. The area size is 2557 × 1787 pixels.

**Figure 5:**
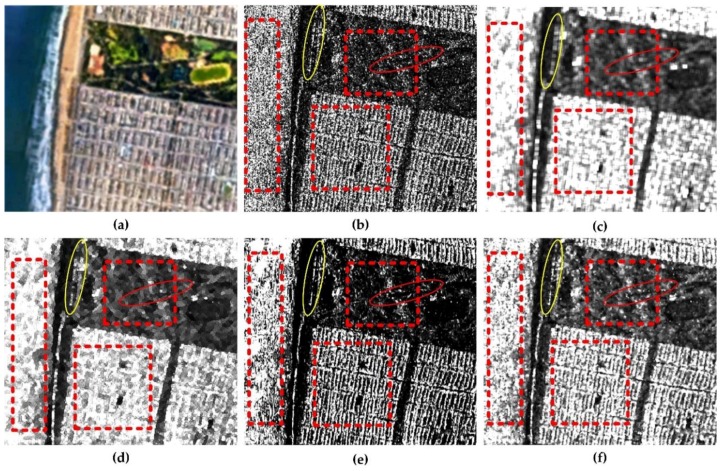
Demonstration and comparison of the effectiveness of different speckle filtering methods for GF-3 span images cropped out from Figure 1b. The area contains 400 × 400 pixels (**a**); Google earth images sampled on 2 September 2017 (**b**); original (**c**); 7 × 7 boxcar (**d**); 7 × 7 Refined Lee filter (**e**); 7 × 7 nonlocal; (**f**) 7 × 7 JRPF.

Since the initial image is too large to exhibit the details of the scene, the texture about city blocks and streets are also not distinct. A part of the scene is cropped out from the left side of Figure 4, which includes the typical land cover. A zoom-in image from the red box in Figure 4 is shown in Figure 5a. From the Google Earth map, the land covers are mainly about vegetation, city blocks, streets, and ocean. The vegetation area is a park with forests, some crook paths and a racecourse. The city blocks contain a large quantity of orderly tilted buildings and streets. The ocean area is on the left of the image, along which are beach and embankment. This small area contains 400 × 400 pixels. 

For comparison, the most commonly used filters of boxcar the Refined Lee filter [35] and the nonlocal filter [23] are applied in the experiment. All the three filters are applied with the 7 × 7 window except for the nonlocal filter. The nonlocal filter is operated in a 21 × 21 searching window with a 7 × 7 patch. The span images of polarimetric data are used to demonstrate the performance of retaining the edge sharpness and texture information as well as speckle reduction. Figure 5b is the original span image, which is smeared by speckle noise severely. Figure 5c shows the span image filtered by 7 × 7 boxcar filter. The result of 7 × 7 boxcar filter exhibits a good characteristic of reducing speckle noise, but the textures of the city blocks are blurred thoroughly. Simultaneously, for its initial deficiency, there exists an inevitable square imprints surrounding the strong targets. For the results of Refined Lee filter in Figure 5d, it seems that the filter has a great power of smoothing the image, and the edges between the park and the city blocks preserved well comparing with the result of boxcar filter. While the street outlines between the city blocks are distorted too much. As for the results of nonlocal and JRPF, both the two filters show strong capability for despeckling. From the visual results, we can find that, not only the speckle is reduced on a large extent, but also the edge signatures are preserved as much as possible with respect to the results of nonlocal filter and JRPF. In terms of edge and texture preservation, the nonlocal shows better performance than other filters in the building area, where we find the city blocks are considerably distinct. While in the areas of volume and the ocean, the speckling result seems discontinuous, i.e., the smoothing results are not as we expect. For a detailed illustration, two typical targets are selected to demonstrate the effectiveness. The targets are a crook path in the park enclosed by a red ellipse and an embankment along the coast enclosed by a yellow ellipse. From Figure 5, we can see that the crook path can only be seen on the results of 7 × 7 nonlocal and 7 × 7 JRPF, and the embankments in both the 7 × 7 nonlocal and JRPF show similar morphological information with the original image. The embankment has the square imprints in the result of 7 × 7 boxcar and discontinuity in the 7 × 7 refined Lee filter in Figure 5d. The streets between the city blocks are all blurred more or less except for the result of 7 × 7 JRPF shown as Figure 5f. From the analysis above, both the nonlocal filter and JRPF shows effective speckle reduction as well as retaining the texture and subtle but distinguishable details, while the nonlocal filter may have better performance in preserving edge signature and texture features.

In order to verify the performance of the speckle noise reduction and edge preservation of different filtering methods, a further quantitative analysis is given by introducing three evaluation parameters. One is the Equivalent Number of Looks (ENL), which is the ratio of the square value of mean to variance of span images over homogeneous areas, and it is the index of the speckle reduction [42]. Higher ENL value indicates higher efficiency in smoothing speckle noise. The second parameter is the Edge Preserving Index (EPI), which is used to test the line and edge preservation of the speckle filtering [43]. The value of EPI is within the range of (0, 1). The higher the EPI value is, the better the performance of the edge preservation is. Another parameter is the coefficient of variation (we use Cx to represent this parameter here) [43], which considers the region heterogeneity. Good texture preservation can be obtained only if the coefficient of variation estimated for the filtered image by means of spatial averages is close to the value expected for the original image. The three parameters are tested mainly on three areas for different filtering results, which are enclosed by the red dash boxes in the ocean area, the vegetation area and the building area, respectively. The parameter values are listed as Table 2.

In Table 2, the ENL, EPI and Cx are calculated before and after filtering by different methods in the three areas aforementioned. For the 7 × 7 boxcar, the ENL are the highest in all the three areas, while the EPI and the Cx are the lowest comparing with the results of other filters. Since the theoretical feature of boxcar, i.e., the theoretical function of boxcar is nothing but an averaging filter, it is predictable that the 7 × 7 boxcar can over smooth the image to some extent. As for the 7 × 7 Refined Lee filter, all the three parameters in the three areas are almost the same with the values of 7 × 7 JRPF. Tiny differences are that the ENL of the 7 × 7 JRPF in the three areas are a little smaller than that of the 7 × 7 refined Lee filter, whereas the EPI are a bit higher. Although the ENL in the 7 × 7 JRPF is comparatively lower than other filters except for 7 × 7 nonlocal, it has a marked increase compared to the original span image. That is to say, the new approach is adequate for the speckle reduction, and also it can preserve the edge feature much better than the other filters beside the nonlocal filter. In terms of the visual speckle filtering results, we see that the nonlocal filter shows a strong performance in speckle reduction and the details preservation. Whereas, the filtering parameters evaluated in the Table 2 show that the results are very abnormal. All the ENL in the three areas are amazingly lower than the original results. Unbelievably, all the EPI are out of the range of (0, 1). Depending on the process procedure of searching a patch in a large window (always 21 × 21), one possible reason is that the filter has thrown away a large amount of coherent information in neighbor pixels and integrated too much information far from the object pixel. Though the results show distinctive edge feature and texture in some scenes, like the city blocks, the land covers after filtering are very dissimilar to the original ones, thus leading to the abnormal results comparative to the general results. For Cx, this filter also has the highest values than other filters, which indicate once more that it can retain the texture very easily.

As for the EPI of ocean area, the value in the 7 × 7 JRPF is higher than the others beside the nonlocal filter. One rational explanation is that beach and ocean waves contribute to the increase of the texture in the sea. For the vegetation area, all the values of ENL are a bit lower than the ocean area in the different filtering methods except for nonlocal filter, while the values of EPI are higher instead. It is obvious that the vegetation area is a park consisted of different targets like the forest and the crook paths. It is hard to say whether it is a homogeneous area. The same tendency is shown in the building area. For the parameter of ENL, the values are much lower, and the values of EPI are much higher in this area. Because this area contains large number of city blocks, and the complex circumstance results in sufficient edge signature and texture information. Thus, the EPI in this area shows a highest value in the result of 7 × 7 JRPF, which can demonstrate that the proposed method has a good performance in the preservation of edge sharpness. In terms of Cx, all the results in the three areas for 7 × 7 JRPF are more close to the Cx of the original span than the other filters, which demonstrates that the 7 × 7 JRPF is very effective in the edge preservation. 

**Figure 6:**
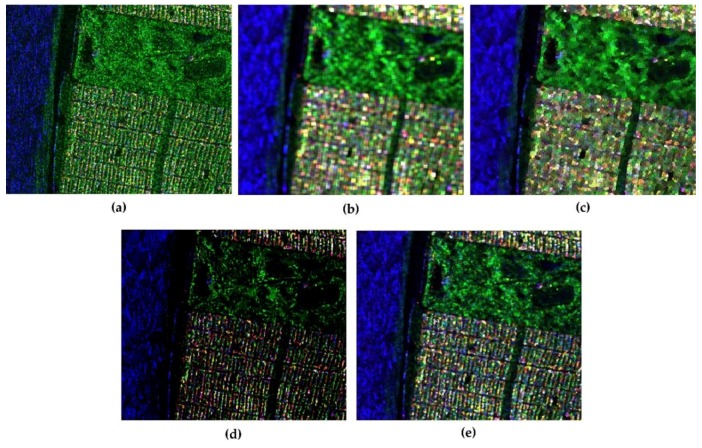
The Pauli decomposition of different speckle filtering methods for GF-3 polarimtric data cropped out from Figure 4. (**a**) original Pauli image without filtering; (**b**) 7 × 7 boxcar; (**c**) 7 × 7 Refined Lee filter; (**d**) 7 × 7 nonlocal; (**e**) 7 × 7 JRPF.

Another remarkable function of JRPF is the preservation of polarimetric scattering mechanism. To describe the performance of retaining polarimetric property, Figure 6 shows the Pauli decompositions with the different filtering methods. In the color composition, red is for $|\mathrm{HH}+\mathrm{VV}{|}^{2}$, green is for $2|{\mathrm{HH}|}^{2}$, and blue is for $|\mathrm{HH}-\mathrm{VV}{|}^{2}$. Corresponding to these colors, the scattering mechanisms are double-bounce, volume, and surface returns, respectively. From the results in e Figure 6a, we can see that the scattering mechanism is badly impaired by the speckle noise, so that the city blocks show a strong performance of volume scattering, which is unfit for its rational surface scattering characteristics. The same impact is on the racecourse in the vegetation area, which is specular return for the scattering mechanism. For comparison, we still use boxcar filter, the Refined Lee filter, the nonlocal filter and JRPF to mitigate the speckle noise. The boxcar is in 7 × 7, the Refined Lee filter in 7 × 7, the nonlocal filter in 7 × 7 (with 21 × 21 searching window) and JRPF in 7 × 7. All the filtering results show reasonable good filtering characteristics of reducing speckle noise and preserving scattering mechanisms. Figure 6d,e illustrate that, in city blocks, the scattering mechanisms of JRPF show more about surface and double-bounce, while the volume is still evident in boxcar (Figure 6b) and the refined Lee filter (Figure 6c). For the specular return, the reflectance in racecourse is much lower in the result of nonlocal and JRPF than that of boxcar and refined Lee filter. All the analyses above verify that the proposed method of speckle filtering is capable of retaining polarimetric scattering mechanism. Alternatively, the Pauli decomposition can also be used to evaluate the edge preservation. From Figure 6, the textures of city blocks and the crook paths in the vegetation area are more distinguishable about nonlocal and JRPF than the other filters. 

**Figure 7:**
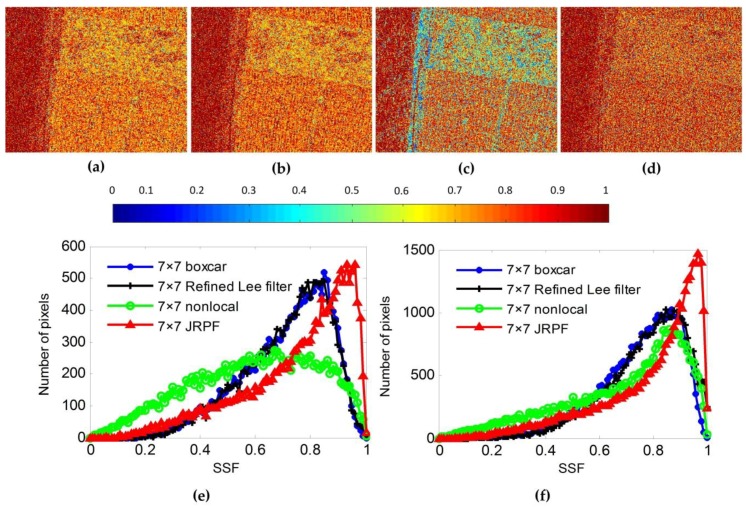
Scattering similarity factor of different speckle filtering methods for GF-3 polarimtric data (**a**) 7 × 7 boxcar; (**b**) 7 × 7 Refined Lee filter; (**c**) 7 × 7 nonlocal; (**d**) 7 × 7 JRPF; (**e**) statistical curves of SSF for different filters in the vegetation areas; (**f**) statistical curves of SSF for different filters in the building areas.

In order to give a further quantitative analysis, Figure 7 shows the polarization similarity measurement after different polarimetric speckle filtering methods. The results are obtained according to the SSF of coherency before and after filtering. It can be seen that, from Figure 7a–d, all the filtering results have high values of SSF in the ocean area. It seems that there is no difference between these filtering methods in the ocean area. Nonetheless, either the SSF in vegetation area or in building area for 7 × 7 boxcar and 7 × 7 refined Lee filter are a bit lower than the result of 7 × 7 JRPF in Figure 7d. The two filters are very similar for the three areas according to the SSF results in Figure 7a,b. Obviously, the SSF of the nonlocal filter shown in Figure 7c exhibit a very different tendency comparative to the former two filters. The SSF values in building area increase a little, while the values decrease too much for most pixels in vegetation area. Though it has the ability to keep the edge and texture very well, some pixels have the SSF values even close to zeros in the result of nonlocal filter, which indicates that this filter cannot keep the scattering mechanisms, especially for the heterogeneous areas. The result can also be explained rationally by the reason we guess aforementioned, that is, the filter brings in too much incoherent information in the process of filtering due to a searching procedure on a large scale. Generally, both in the vegetation area and in the building area, the value of SSF can be improved largely for the new proposed method. Compared with the results of the building area in JRPF, the SSF in the vegetation area increases more significantly. All the analysis above, we find that only the method of JRPF can preserve the scattering mechanisms very well, at least for the three common scattering mechanisms (double-bounce, volume, and surface scattering).

To visualize the performance, we obtain statistical characteristics of polarization similarity. Four different statistical curves of SSF under different filtering methods in both vegetation area and building area are presented in Figure 7e,f, respectively. Among the color, blue represents the statistical curve filtered by 7 × 7 boxcar, black represents by 7 × 7 refined Lee filter, green represents by 7 × 7 nonlocal, and the red color is filtered under 7 × 7 JRPF. From all the statistical results, it can be seen that the SSF curves of the 7 × 7 boxcar and black 7 × 7 refined Lee filter are almost in superposition and their peaks are around 0.8 in vegetation area. The curve of nonlocal does not have an obvious peak with the low SSF in this area. Whereas, the peak of JPRF is near to 0.9, which shows that the new method can maintain the polarization scattering characteristics better. Similarly, the SSF in the building area are selected for statistics, and peaks of SSF curves of the other three filtering methods comparative to JPRF are around 0.88. The peak of SSF curves after filtering by JPRF is close to 0.97. In other words, the speckle filtering method of JPRF can almost maintain the polarization identically with its original one in the building area. 

**Figure 8:**
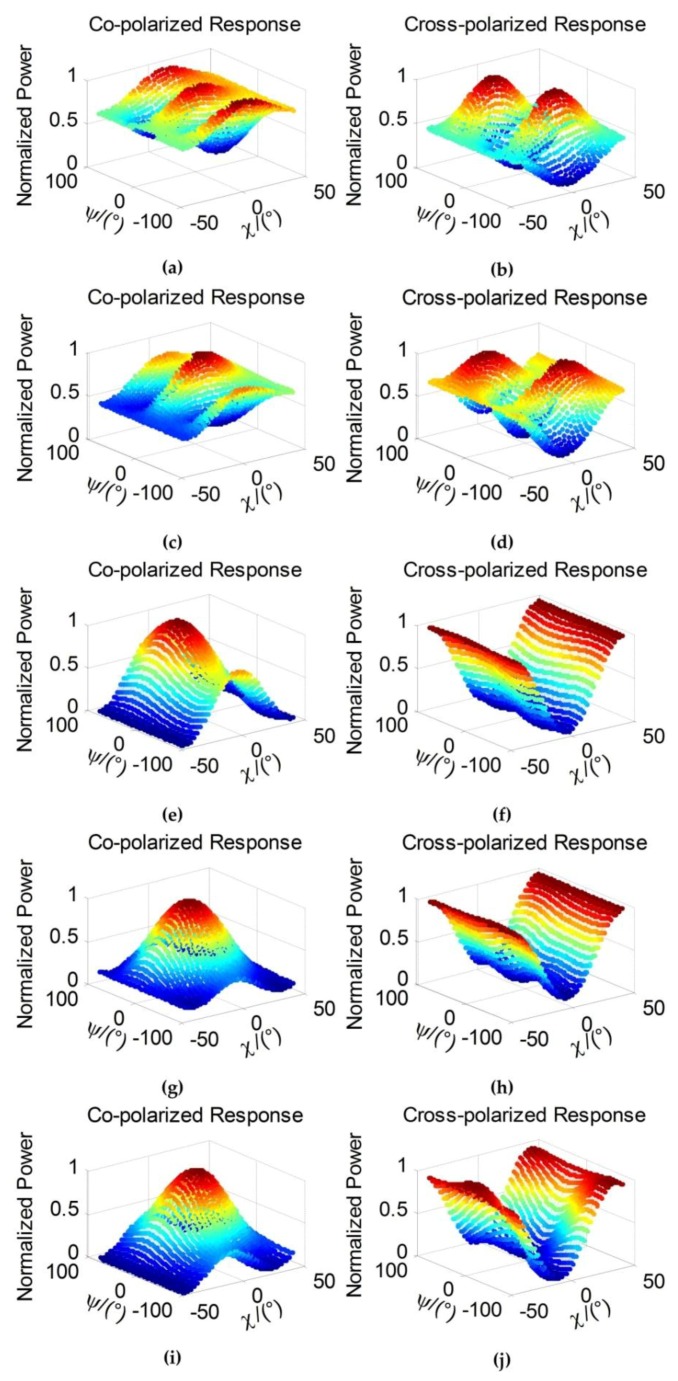
Co-polarized and cross-polarized signatures of (115, 342) pixel in building area of Figure 5. (**a**,**b**) 7 × 7 boxcar; (**c**,**d**) 7 × 7 Refined Lee filter; (**e**,**f**) 7 × 7 nonlocal; (**g**,**h**) 7 × 7 JRPF; (**i**,**j**) original.

**Figure 9:**
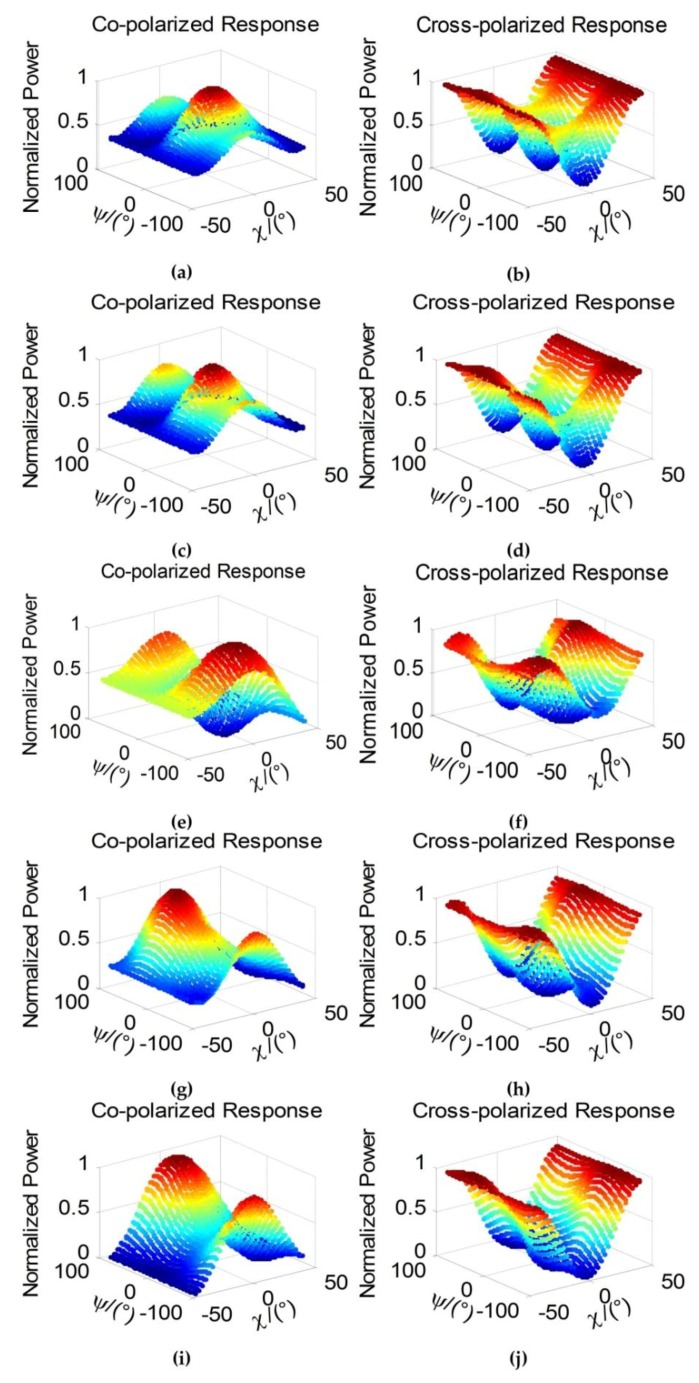
Co-polarized and cross-polarized signatures of (358, 275) pixel in building area of Figure 5. (**a**,**b**) 7 × 7 boxcar; (**c**,**d**) 7 × 7 Refined Lee filter;(**e**,**f**) 7 × 7 nonlocal; (**g**,*h*) 7 × 7 JRPF; (**i**,**j**) original.

Furthermore, we pick out two strong point target scatterers in vegetation area and building area to explore their polarization response separately. The polarization response shows the normalized power at different polarized patterns [43]. The co-polarized and cross-polarized response, which are formed in a combination of ellipse orientation angle ψ (degree) and ellipticity angle χ (degree), are applied to study the polarization signature. In Figure 8, the co-polarization and cross-polarization signatures of pixel (115, 342) in vegetation area are described to illustrate the performance of retaining polarization property. From the left side to the right side of Figure 8, the results are about 7 × 7 boxcar, 7 × 7 refined Lee filter, 7 × 7 nonlocal filter (with 21 × 21 searching window), 7 × 7 JRPF and the original response without filtering. The upside response of Figure 8 is co-polarized and the down side cross-polarized. The contrast of these methods indicates that the JRPF can retain the similar response with the original one, which demonstrates a good performance of the new speckle filtering. An identical process is operated in the pixel (358, 275) in building area as the results in Figure 9. All the results of polarization response are distorted severely corresponding to the original one, apart from the result of JRPF.

**Figure 10:**
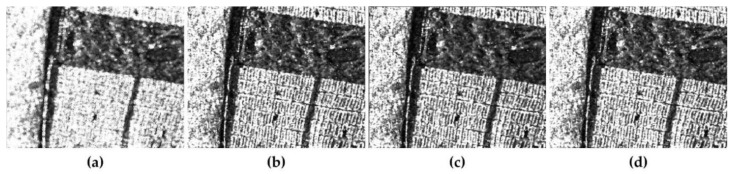
Comparison of speckle filtering results by different combination of the three principles for GF-3 span images (**a**) MCPF&SMPF; (**b**) MCPF&SCPF; (**c**) SCPF&SMPF; (**d**) JRPF.

For the sake of argument, we intend to discuss all the possible results by ignoring one of the three principles. The processing procedure is still the same as the flowchart shown as Figure 3. The only difference is to ignore one principle shown in the colored boxes in Figure 3. Figure 10 shows the speckle filtering results by different combination of the three principles. Figure 10a is the result of combination of MCPF and SMPF by ignoring SCPF. Figure 10b is the result of combination of MCPF and SCPF by ignoring SMPF, and Figure 10c is the result of combination of the other two principles. The JRPF result is present as a contrast in Figured. When only consider the principles of morphological consistency and scattering mechanism preservation, the filtered span image depicts a bad performance in preserving the edge signature compared with the other combination results, especially in the city blocks (see Figure 10a). However, the other two forms of combination can preserve the texture information very well (see Figure 10b,c), and also the difference seems indistinctive between the two combination results and the JRPF result. It suggests that the statistic characteristic principle plays an important role in the performance of preserving the edge signature and texture features. To investigate whether the two combinations can satisfy the requirement of the speckle filtering with the same effectiveness as JRPF, the ENL, EPI and Cx values after filtering by two combination principles in the three areas are given in Table 3. 

**Figure 11:**
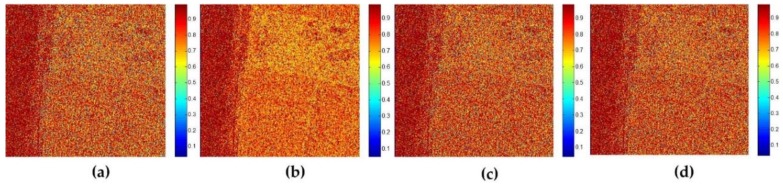
The SSF of speckle filtering results by different combination of the three principles for GF-3 polarimtric data (**a**) MCPF&SMPF; (**b**) MCPF&SCPF; (**c**) SCPF&SMPF; (**d**) JRPF.

In Table 3, the ENL, EPI and Cx values after filtered by different combination of the three principles in different areas are listed. We find that the ENL value and the EPI value are in an opposite trend in vegetation area except for the combination of MCPF and SMPF. The combination of SCPF and SMPF has the highest value of ENL in the vegetation area, while the value of EPI is the lowest for this combination. For the combination of MCPF and SMPF, with a very similar ENL value of the combination of SCPF and SMPF, there comes out an abnormal value of EPI, i.e., both the value of ENL and EPI are very high comparative to the value of others. One possible reason is that the morphological windows express an obvious feature for the edge preservation. Though the result seems to be what we expect, the visual result in Figure 10a shows blur especially in the creek path. For the combination of MCPF and SCPF, the ENL value decreases a little, whereas the EPI increases. Furthermore, JRPF has a lower value of ENL and higher value of EPI than the combination results of MCPF and SCPF. Also, the two values in the combination of MCPF and SCPF are very close to the values in the JRPF. Accordingly, we can only conclude that it is very efficiency in speckle reduction for the combination of SCPF and SMPF, while the JRPF has a better result than the other three filtering forms for edge preservation in the vegetation area. As for the building area, the values of ENL in the four filtering forms are much lower than the corresponding results in vegetation area, and the values of EPI are a bit higher except for the combination of MCPF and SMPF. Amazingly, the ENL value and the EPI value are in the same trend in building area excluding the value of MCPF and SMPF. Though the ENL value is highest for combination of MCPF and SMPF, the EPI is the lowest amongst the four filtering forms. Whereas, the EPI value of JRPF is highest with an acceptable value of ENL. Moreover, the results for the combination of MCPF and SCPF are very similar to the values of JRPF. Considering abundant texture, we believe that JRPF keep a better performance than the others. In the ocean area, the value s of ENL and EPI are close to the values in vegetation area and the tendency is the same except for the combination of MCPF and SMPF. With a highest value of ENL and lowest EPI, we may think that this combination is a better strategy for filtering when only consider about the performance for ocean area. As a matter of fact, when talking about the speckle filtering, we may concern more about the vegetation area and city blocks which include more scattering information. In terms of the speckle reduction and the edge preservation for the whole image, the filtering method of combination of MCPF and SCPF seems to be capable of substituting the JRPF. Nevertheless, the scattering mechanism preservation should be also taken into consideration in the speckle filtering. For comparison, the SSF of filtering results by different combination of the three principles are shown in Figure 11. As for the parameter of Cx, Whatever the region is, the values of Cx are very close to each other for different combination of the principles. The main features are that the values for different combination of principles are much higher in the building area and the values for JRPF are always the highest of all the combinations of the principles in all the three areas.

In Figure 11, we can see that the SSF values in both the vegetation area and building area for the combination of MCPF and SCPF are much lower than that for the other combination form, as well as the result in JRPF. The other three filtering forms are very similar for the value of SSF, because only the combination of MCPF and SCPF neglect the restriction of scattering mechanism preservation. That is to say this combination cannot substitute the JRPF at all. From above all, none of the three principles aforementioned could be ignored during the speckle filtering for polarimetric SAR data.

**Figure 12:**
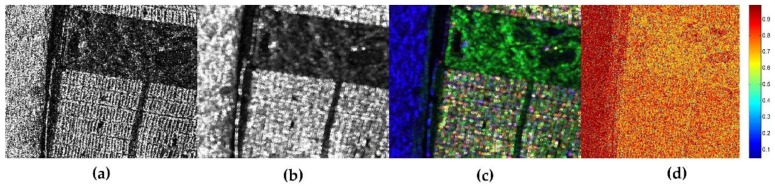
The speckle filtering results only by MCPF for GF-3 polarimtric data (**a**) original; (**b**) MCPF result; (**c**) Pauli decomposition; (**d**) SSF.

**Figure 13:**
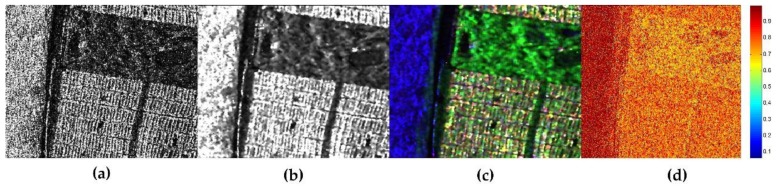
The speckle filtering results only by SCPF for GF-3 polarimtric data (**a**) original; (**b**) SCPF result; (**c**) Pauli decomposition; (**d**) SSF.

**Figure 14:**
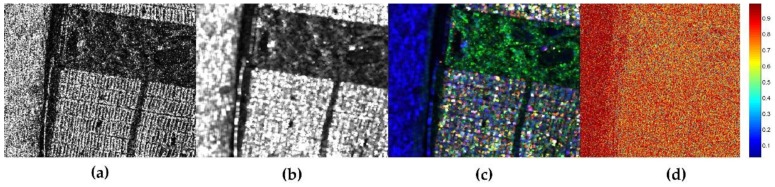
The speckle filtering results only by SMPF for GF-3 polarimtric data (**a**) original; (**b**) SMPF result; (**c**) Pauli decomposition; (**d**) SSF.

To explore the contribution of each stage of the three restrict principles (MCPF/SMPF/SCPF), we perform additional experiments with only one of the three principles. The filtering results in Figure 12, Figure 13 and Figure 14 are for MCPF, SCPF and SMPF, respectively. All these figures include four images, which are the original span image, the filtered span image, the Pauli decomposition and the SSF after filtering. From Figure 12, we can see that the MCPF have reduced the speckle noise on a large extent. A very obvious feature is that this filter keeps the most objects have a camber or linear brim for its restriction principle, which is very adaptive to the edge signature especially in the vegetation area (like most trees). The main contribution of this stage is that it can avoid the square imprints which may always emerge in the boxcar filter, though the edge preservation may do not perform very well under this principle. For the filtering results only by SCPF, Figure 13 shows that this principle can reduce the speckle noise more thoroughly than the filtering result only by MCPF. Obviously, this restriction can keep the edge and texture as much as possible. Though there may be some small square imprints in the vegetation area, the edge signature is very distinctive, especially in building area. As for the result filtered only by SMPF, a dominant contribution is that it can preserve the polarimetic scattering mechanism for all the three different areas. From Figure 14c, we can see that the volume scatterers are much less and the surface and double-bounce scatterers are much more comparable to the results of Figure 12c and Figure 13c in building area. Also, the SSF are much higher than the other two restriction principles.

## 5. Conclusions

Speckle filtering is a very essential procedure for PolSAR data in most applications. Because scattering characteristics are obtained by second order moments estimation of multi-polarization data, which is the process of averaging coherency and covariance matrices from neighboring pixels. The speckle noise is induced by waves’ initial scattering phenomenon from many elementary scatterers. In order to get high accuracy of covariance or coherency matrices, several restriction principles are taken into consideration. The morphological consistency, the statistic characteristics restriction and the scattering mechanism preservation are treated as the main principles. To preserve the edge signature or the texture of the scene, a new template, which is more suitable for the point targets with cambered brims or the line target with linear brims, is designed to ensure the morphological consistency. As for the statistic characteristics restriction, the extent sigma filter is used to restrict the pixels in the template. At last, a polarimetric similarity factor is applied to the pixels to guarantee the polarimertic scattering mechanism stay unchangeable. In this paper, we name this new procedure of PolSAR speckle filtering as JRPF (joint restriction principles filter) for single-channel SAR images. The effectiveness of the proposed filter was demonstrated using GF3 polarimetric data and compared favorably with the most common used filters of boxcar and the refined Lee filter. The advantage of the proposed filter rests at keeping the image sharpness and preserving the scattering mechanism as well as speckle reduction. Further, though the JRPF can keep the polarimetric property as same as the original one, the polarization response of a strong point targets may presents an uncommon mechanism signature. One possible reason is that there may still have residual distortion. To cope with this issue, another important preprocessing, which is called polarization calibration, needs to be studied in the next step. For further argument, the possible filtering results by ignoring one of the three principles are discussed. The results show that the combination of MCPF and SCPF is capable of substituting the JRPF in terms of the speckle reduction and the edge preservation, while none of the three restriction principles could be ignored during the speckle filtering of polarimetric SAR data for the requirement described in the paper. In this paper, we restrict our experiment for GF-3 SAR data. Because that we are not sure whether this algorithm can keep the same features for the polarimetric SAR data implemented by other SAR satellites or not, though the method shows good performance on speckle reduction and edge retaining as well as scattering preservation on GF-3 polarimetric SAR data. Actually, each speckle filtering algorithm has its merit in speckle level reduction under a unique circumstance and deficiency of blurring the images in other conditions. The effectiveness of filters is determined by the scenes of application and, to some extent, the parameters of acquisition of SAR systems. Whether the new method can keep the same features in implementation of other SAR satellites, we regard this as an interesting and hopeful study in our future work.

## Figures and Tables

**Table 1:** Sigma range (*I*_1_*, I*_2_) for one-look intensity data was computed based on the speckle pdfs and the speckle noise standard deviation ${\tilde{\eta }}_{v}$ is for MMSE application [21].

Sigma ξ	$I_{1}$	$I_{2}$	${\tilde{\eta }}_{v}$
0.50	0.436	1.920	0.4057
0.60	0.343	2.210	0.4954
0.70	0.254	2.582	0.5911
0.80	0.168	3.094	0.6966
0.90	0.084	3.941	0.8191
0.95	0.043	4.840	0.8599

**Table 2:** ENL, EPI and Cx values before and after filtering by different methods in the ocean area, the vegetation area and the building area.

		Original Span	7 × 7 Boxcar	7 × 7 Refined Lee Filter	7 × 7 Nonlocal	7 × 7 JRPF
Ocean	ENL	0.799	10.524	5.919	0.314	4.891
	EPI	1.000	0.107	0.232	1.135	0.350
	Cx	1.071	0.3083	0.384	1.782	0.557
Vegetation	ENL	0.867	5.774	4.263	0.251	3.590
	EPI	1.000	0.140	0.300	0.317	0.499
	Cx	1.017	0.416	0.484	1.991	0.602
building	ENL	0.173	2.804	0.808	0.003	0.348
	EPI	1.000	0.141	0.347	1.923	0.543
	Cx	1.904	0.597	1.112	21.060	1.732

**Table 3:** ENL, EPI and Cx values after filtered by different combination of the three principles in different areas.

		MCPF&SMPF	MCPF&SCPF	SCPF&SMPF	JRPF
Vegetation	ENL	4.038	3.775	4.245	3.590
	EPI	0.597	0.445	0.343	0.499
	Cx	0.573	0.600	0.555	0.602
building	ENL	0.782	0.333	0.291	0.348
	EPI	0.392	0.530	0.455	0.543
	Cx	1.130	1.732	1.653	1.732
ocean	ENL	5.110	3.783	4.978	4.891
	EPI	0.261	0.345	0.285	0.350
	Cx	0.404	0.557	0.478	0.557
